# Dense dislocations enable high-performance PbSe thermoelectric at low-medium temperatures

**DOI:** 10.1038/s41467-022-34227-3

**Published:** 2022-10-28

**Authors:** Liqing Xu, Yu Xiao, Sining Wang, Bo Cui, Di Wu, Xiangdong Ding, Li-Dong Zhao

**Affiliations:** 1 State Key Laboratory for Mechanical Behavior of Materials, Xi’an Jiaotong University, 710049 Xi’an, China; 2 School of Materials Science and Engineering, Beihang University, 100191 Beiijng, China; 3 Institute of Nuclear Physics and Chemistry, China Academy of Engineering Physics, 621900 Mianyang, China; 4 School of Materials Science and Engineering, Shaanxi Normal University, 710049 Xi’an, China

**Keywords:** Materials science, Energy harvesting

## Abstract

PbSe-based thermoelectric materials exhibit promising *ZT* values at medium temperature, but its near-room-temperature thermoelectric properties are overlooked, thus restricting its average *ZT* (*ZT*_ave_) value at low-medium temperatures. Here, a high *ZT*_ave_ of 0.90 at low temperature (300–573 K) is reported in *n*-type PbSe-based thermoelectric material (Pb_1.02_Se_0.72_Te_0.20_S_0.08_−0.3%Cu), resulting in a large *ZT*_ave_ of 0.96 at low-medium temperatures (300–773 K). This high thermoelectric performance stems from its ultralow lattice thermal conductivity caused by dense dislocations through heavy Te/S alloying and Cu interstitial doping. The dislocation density evaluated by modified Williamson-Hall method reaches up to 5.4 × 10^16^ m^−2^ in Pb_1.02_Se_0.72_Te_0.20_S_0.08_−0.3%Cu. Moreover, the microstructure observation further uncloses two kinds of dislocations, namely screw and edge dislocations, with several to hundreds of nanometers scale in length. These dislocations in lattice can strongly intensify phonon scattering to minimize the lattice thermal conductivity and simultaneously maintain high carrier transport. As a result, with the reduced lattice thermal conductivity and optimized power factor in Pb_1.02_Se_0.72_Te_0.20_S_0.08_−0.3%Cu, its near-room-temperature thermoelectric performance is largely enhanced and exceeds previous PbSe-based thermoelectric materials.

## Abstract (web summary)

Thermoelectric material is capable of realizing direct conversion between heat and electricity. Here, the authors obtain high thermoelectric performance in PbSe at low-medium temperatures by importing dense dislocations.

## Introduction

Thermoelectric (TE) materials have the ability to realize direct conversion between waste heat and electricity, which can ease the energy and environment crisis^1–4^. The energy conversion efficiency of thermoelectric devices depends on the dimensionless figure of merit (*ZT*), *ZT* = *S*^2^*σT*/*κ*_tot_, where *S*, *σ*, *T*, *κ*_tot_ denote Seebeck coefficient, electrical conductivity, absolute temperature in kelvin and the total thermal conductivity, respectively. Notably, the key to develop high-performance TE materials is decoupling these inter-dependent TE parameters to improve power factor *PF* = *S*^2^*σ* or reduce *κ*_tot_, where *κ*_tot_ is a sum of electronic thermal conductivity (*κ*_ele_) and lattice thermal conductivity (*κ*_lat_)^5–7^.

Lead chalcogenides are good thermoelectric materials, and many advanced strategies to optimize thermoelectric performance have been developed based on lead chalcogenides, especially for PbTe, such as band convergence^8,9^, resonant state^10,11^, nanostructuring^12–16^, etc. Among lead chalcogenides, PbSe thermoelectric material is a promising alternative of PbTe due to its low-cost Se element and balanced carrier and phonon transport properties. To enhance the thermoelectric performance in PbSe, some effective approaches have recently been reported, including band flattening to improve carrier effective mass^17–19^, band sharpening to tune carrier mobility^20,21^, dynamic doping to optimize carrier density^20,22^, defects design to scatter phonon transport^23–25^, etc. Notably, it is found that the vacancy- or interstitial-induced dislocations in PbSe-based thermoelectric material can largely reduce the lattice thermal conductivity while causing little effect on electrical transport properties, thus finally enhancing the peak *ZT* value. However, the dislocation strategies in previous works fail to optimize the near-room-temperature thermoelectric performance, and its average *ZT* value at low-medium temperatures maintains at low value, which might be caused by the low dislocation density to intensify phonon scattering at low temperature range. Therefore, to further enhance the thermoelectric performance in PbSe at low-medium temperatures, this work aims to produce dense dislocations in PbSe lattice in order to substantially suppress the lattice thermal conductivity.

In fact, the dislocation produced by only vacancy^26–28^ or interstitial^22,29,30^ is limited to their solubility in matrix, which makes it challengeable to achieve high dislocation density (*N*_D_ > 10^15^ m^−2^) in thermoelectric materials. Herein, this work firstly introduces heavily Te and S alloying in PbSe matrix so as to cause strong lattice distortion, which can provide extra energy for dislocation formation. And then, based on the optimal Te and S co-alloyed Pb_1.02_Se_0.72_Te_0.20_S_0.08_ sample, the Cu interstitial doping is imported to accelerate dislocation formation. With these two successive steps, very high dislocation density of 5.4 × 10^16^ m^−2^ can be obtained in Pb_1.02_Se_0.72_Te_0.20_S_0.08_−0.3%Cu, which is much higher than that in previously reported *n*-type PbSe-based samples. Such dense dislocations in Pb_1.02_Se_0.72_Te_0.20_S_0.08_−0.3%Cu contributes to ultralow lattice thermal conductivity in the whole-temperature range. Moreover, its room-temperature lattice thermal conductivity can be reduced to 0.42 W m^−1^ K^−1^ and the minimal lattice thermal conductivity reaches at 0.29 W m^−1^ K^−1^ at 573 K. Additionally, these dislocations in matrix play slight impact on carrier transport and can maintain a decent weighted carrier mobility. Eventually, the thermoelectric performance is substantially enhanced in Pb_1.02_Se_0.72_Te_0.20_S_0.08_-*x*%Cu (*x* = 0–0.40) at low-medium temperatures, and a room-temperature *ZT* value of 0.62 and *ZT*_ave_ of 0.90 at 300–573 K can be realized in Pb_1.02_Se_0.72_Te_0.20_S_0.08_−0.3%Cu.

## Results

To obtained high thermoelectric performance in *n*-type PbSe-based material at low-medium temperatures, this work aims to import dense dislocations to substantially reduce the lattice thermal conductivity. Firstly, over-stoichiometric Pb atoms are used to realize electron dominated *n*-type Pb_1.02_Se. Then, Te and S alloying in Pb_1.02_Se are following introduced to cause lattice distortion, which is favorable for dislocation formation, and also results in strong strain field to reduce lattice thermal conductivity. Finally, Cu interstitial doping in Pb_1.02_Se_0.72_Te_0.20_S_0.08_ promotes the dislocation formation, and high dislocation density in Pb_1.02_Se_0.72_Te_0.20_S_0.08_−0.3%Cu is revealed by microstructure observation. These dense dislocations largely reduce the lattice thermal conductivity and lead to high thermoelectric performance at low-medium temperatures in *n*-type Pb_1.02_Se_0.72_Te_0.20_S_0.08_−0.3%Cu.

### Crystal structure identification and lattice thermal conductivity

**Fig. 1:**
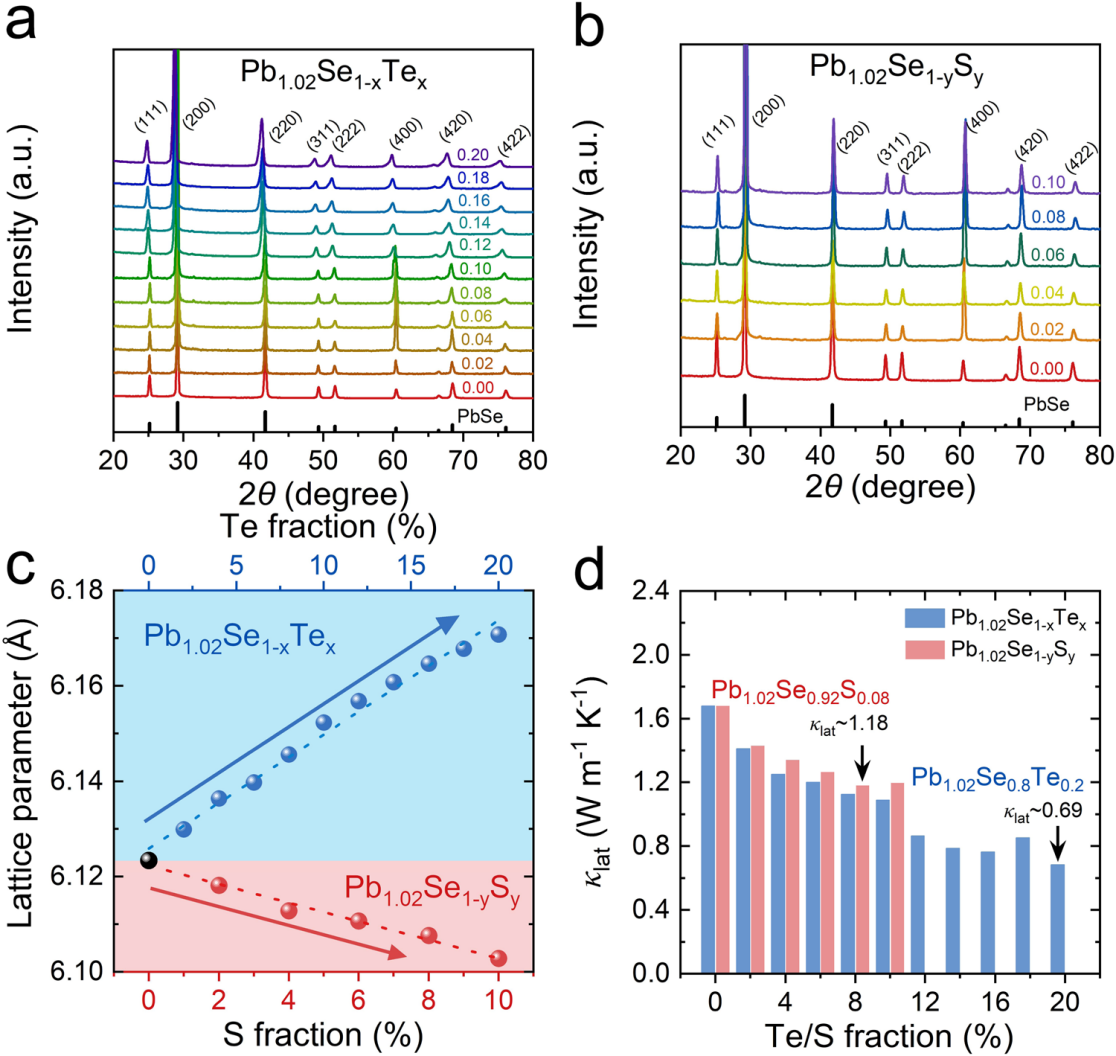
PXRD and lattice thermal conductivity of Pb_1.02_Se_1–*x*_Te_*x*_ and Pb_1.02_Se_1–*y*_S_*y*_. **a** Powder XRD patterns of Pb_1.02_Se_1–*x*_Te_*x*_ (*x* = 0–0.20). **b** Powder XRD patterns of Pb_1.02_Se_1–*y*_S_*y*_ (*y* = 0–0.10). **c** Lattice parameter as a function of Te/S fraction. **d** Room-temperature lattice thermal conductivity as a function of Te/S fraction in Pb_1.02_Se_1–*x*_Te_*x*_ (*x* = 0–0.20) and Pb_1.02_Se_1–*y*_S_*y*_ (*y* = 0–0.10).

The powder XRD patterns of Pb_1.02_Se_1–*x*_Te_*x*_ (*x* = 0–0.20) and Pb_1.02_Se_1–*y*_S_*y*_ (*y* = 0–0.10) can be completely indexed as the rock-salt structure (Fig. 1a, b), and with rising Te/S alloying content, lattice expansion and contraction are clearly observed in Te-alloyed PbSe and S-alloyed PbSe samples, respectively. Moreover, the lattice parameter of 6.12 Å in Pb_1.02_Se linearly increases to 6.17 Å in Pb_1.02_Se_0.8_Te_0.2_ and decreases to 6.10 Å in Pb_1.02_Se_0.9_S_0.1_ (Fig. 1c). As a result, the room-temperature lattice thermal conductivity of Pb_1.02_Se_1–*x*_Te_*x*_ (*x* = 0–0.20) and Pb_1.02_Se_1–*y*_S_*y*_ (*y* = 0–0.10) show significant decrease from 1.68 W m^−1^ K^−1^ in Pb_1.02_Se to 0.69 W m^−1^ K^−1^ in Pb_1.02_Se_0.8_Te_0.2_ and 1.18 W m^−1^ K^−1^ in Pb_1.02_Se_0.92_S_0.08_ (Fig. 1d). The origin of diminished lattice thermal conductivity in Pb_1.02_Se_1–*x*_Te_*x*_ (*x* = 0–0.20) and Pb_1.02_Se_1-y_S_y_ (*y* = 0–0.10) stems from the lattice distortion after alloying, which can intensify phonon scattering due to mass and strain field fluctuations in matrix lattice. As the heavy Te/S alloying in *n*-type PbSe not only scatters phonon but also blocks carrier transport, no enhancement of *ZT* value can be obtained in Pb_1.02_Se_1–*x*_Te_*x*_ (*x* = 0–0.20) and Pb_1.02_Se_1–*y*_S_*y*_ (*y* = 0–0.10) samples (Supplementary Fig. 1–4).

**Fig. 2:**
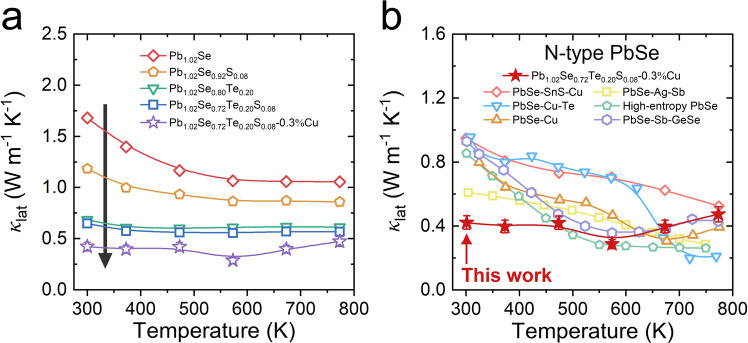
Optimization and comparison of lattice thermal conductivity. **a** Optimization of temperature-dependent lattice thermal conductivity from Pb_1.02_Se to Pb_1.02_Se_0.72_Te_0.20_S_0.08_−0.3%Cu. **b** Comparison of lattice thermal conductivity in *n*-type PbSe. Error bars are ±10%.

Based on the minimal thermal conductivity in Te/S-alloyed PbSe, Cu interstitial doping is introduced into Pb_1.02_Se_0.72_Te_0.20_S_0.08_. With these successive steps, the lattice thermal conductivity in this work is largely suppressed (Fig. 2a). The room-temperature lattice thermal conductivity is suppressed from 1.68 W m^−1^ K^−1^ in Pb_1.02_Se to 0.65 W m^−1^ K^−1^ in Pb_1.02_Se_0.72_Te_0.20_S_0.08_, and further decreased to 0.42 W m^−1^ K^−1^ in Pb_1.02_Se_0.72_Te_0.20_S_0.08_−0.3%Cu. Moreover, the temperature-dependent lattice thermal conductivity in Pb_1.02_Se_0.72_Te_0.20_S_0.08_−0.3%Cu can maintain at very low value (< 0.5 W m^−1^ K^−1^) in the whole-temperature range (Fig. 2b), which is comparable with other advanced *n*-type PbSe, especially at 300–573 K, including PbSe-SnS-Cu^20^, PbSe-Ag-Sb^31^, PbSe-Cu-Te^30^, high-entropy PbSe^32^, PbSe-Cu^22^, PbSe-Sb-GeSe^24^. It is worth noting that the theoretical limit of lattice thermal conductivity in PbSe compound is 0.38 W m^−1^ K^−1^ based on the assumption of minimal phonon mean free path^24,33^. However, some lower lattice thermal conductivity can also be obtained in experimental results, especially in Cu-contained samples, which is closely related to the abnormal Cu atom diffusion with increasing temperature. In fact, some Ag/Cu-based compounds can achieve ultralow lattice thermal conductivity due to the highly mobile Ag/Cu atoms^34^, indicating that evaluating the theoretical lattice thermal conductivity should further consider the roles of atom diffusion in thermoelectric materials^35^.

### Dislocation density estimation with modified Williamson-Hall (MWH) method

**Fig. 3:**
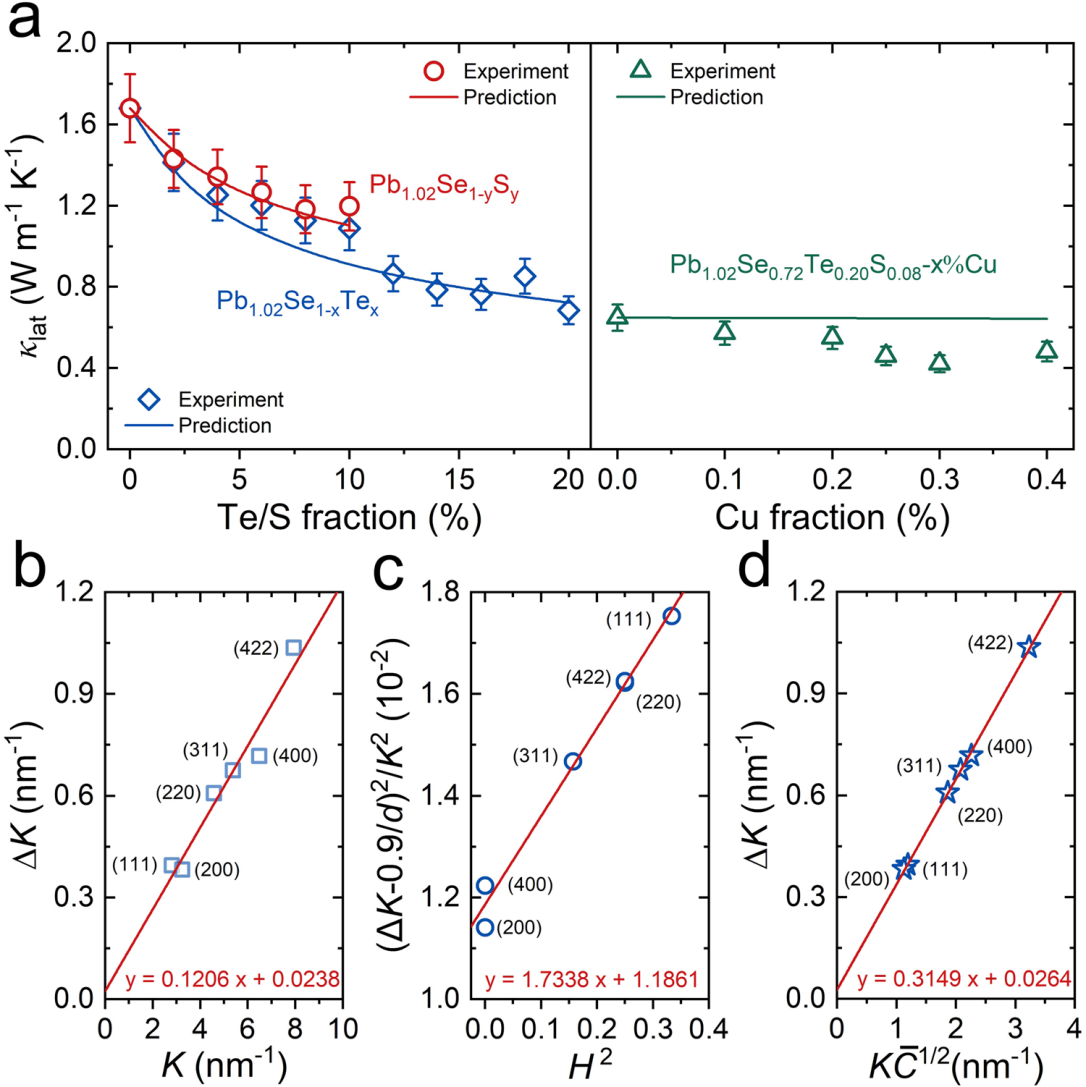
Lattice thermal conductivity of model prediction and estimation of dislocation density in Pb_1.02_Se_0.72_Te_0.20_S_0.08_−0.3%Cu by MWH method. **a** Room-temperature lattice thermal conductivity as a function of Te, S, and Cu content for Pb_1.02_Se_1–*x*_Te_*x*_ (*x* = 0.20), Pb_1.02_Se_1–*y*_S_*y*_ (*y* = 0–0.10), and Pb_1.02_Se_0.72_Te_0.20_S_0.08_-*x*%Cu (*x* = 0–0.40). The solid curve is the prediction by Callaway model. Error bars are ±10%. **b** Plot of Δ*K* ~ *K*. **c** Plot of (Δ*K*−0.9/*d*)/*K*^2^ ~ *H*^2^. **d** Plot of Δ*K* ~ $K\overline{C}$^1/2^.

After Cu interstitial doping in Pb_1.02_Se_0.72_Te_0.20_S_0.08_-*x*%Cu (*x* = 0–0.40), all samples still maintain cubic phase and the lattice slightly expands with increasing Cu content, proving the Cu interstitials in matrix (Supplementary Fig. 5). The room-temperature lattice thermal conductivity in Pb_1.02_Se_1–*x*_Te_*x*_ (*x* = 0–0.2) and Pb_1.02_Se_1–*x*_S_*x*_ (*x* = 0–0.1) can well follow the predicted value by Callaway model (Fig. 3a and Supplementary Table 1). After Cu interstitial doping, the lattice thermal conductivity continuously decreases and begins to deviate from the predicted value. The large deviation of lattice thermal conductivity between experiment result and predicted value suggests extra effects on phonon scattering. As Cu interstitials incline to aggregate and could form line defects in matrix, the modified Williamson-Hall (MWH) method is used to estimate dislocation density. Based on XRD diffraction peak broadening effect, the MWH method for calculating dislocation density derived from Ungár and Borbély can be written as follows when ignore the non-interpreted high-order error terms *O*^36–38^:

1 $$\Delta K=0.9/d+{(\pi {A}^{2}{b}^{2}/2)}^{1/2}{N}_{D}^{1/2}(K{\overline{C}}^{1/2})$$

where $K=2\,\sin \theta /\lambda$, $\Delta K=2\,\cos \theta (\varDelta \theta )/\lambda$, *θ* and *λ* are the diffraction angle and the wavelength of X-rays, *d* is the average grain size, *b* is the magnitude of Burgers vector and *N*_D_ is dislocation density. The derivation of the equation is shown in Supporting Information.

The estimation of dislocation density is divided into three steps. First, the line profiles were fitted by Gaussian function method to obtain the exact Bragg position and full width at half maximum (FWHM). Six diffraction angles are selected for *K* ~ Δ*K* plot (Fig. 3b), and corresponding indices of crystal plane and FWHM are listed in Supplementary Table 2. The value of 0.9/*d* is obtained by linear fitting. Then, calculating the value of (Δ*K*−0.9/*d*)^2^/*K*^2^, and the value of *q* is obtained by fitting formular of (Δ*K*−0.9/*d*)^2^/*K*^2^ ~*H*^2^ as follows:

2 $$\frac{{(\Delta K -0.9/d)}^{2}}{{K}^{2}}=(\pi {A}^{2}{b}^{2}/2){N}_{D}{\overline{C}}_{h00}(1-{{{qH}}}^{2})$$

where the calculated *q* equals −1.46 (Fig. 3c). The $\overline{C}$ of different crystal plane is obtained by equation (S29). Finally, the dislocation density can be calculated by the slope of the Δ*K* ~ $K\overline{C}$^1/2^ plot in Eq. (1) (Fig. 3d), the details of other parameters are listed in Supplementary Table 3. Moreover, the final dislocation density in Pb_1.02_Se_0.72_Te_0.20_S_0.08_−0.3%Cu can reach up to 5.4 × 10^16^ m^−2^, which is much higher than that in other lead chalcogenides, such as *N*_D_ of 5 × 10^12 ^m^−2^ in *n*-type Pb_0.95_Sb_0.33_Se^26^, *N*_D_ of 3 × 10^15^ m^−2^ in *n*-type Pb_0.95_Sb_0.33_Se_0.6_Te_0.4_^39^, *N*_D_ of 4 × 10^12 ^m^−2^ in *p*-type Na_0.025_Eu_0.03_Pb_0.945_Te^40^, and *N*_D_ of 2 × 10^14 ^m^−2^ (grain interior) and 1 × 10^16 ^m^−2^ (grain boundary) in PbTe-Ag^41^. The high dislocation density in Pb_1.02_Se_0.72_Te_0.20_S_0.08_−0.3%Cu is ascribed to the strong lattice distortion caused by heavy Te/S alloying and Cu interstitial doping. Compared with Pb_1.02_Se_0.72_Te_0.20_S_0.08_ sample (Supplementary Table 2 and Supplementary Fig. 6), Pb_1.02_Se_0.72_Te_0.20_S_0.08_−0.3%Cu owns larger value of FWHM, thus resulting in higher dislocation density. Additionally, this result indicates that Cu interstitial can further promote dislocation formation and indirectly proves a lower dislocation formation energy in Pb_1.02_Se_0.72_Te_0.20_S_0.08_−0.3%Cu than that in Pb_1.02_Se_0.72_Te_0.20_S_0.08_.

### Microstructure observations in Pb_1.02_Se_0.72_Te_0.20_S_0.08_−0.3%Cu

**Fig. 4:**
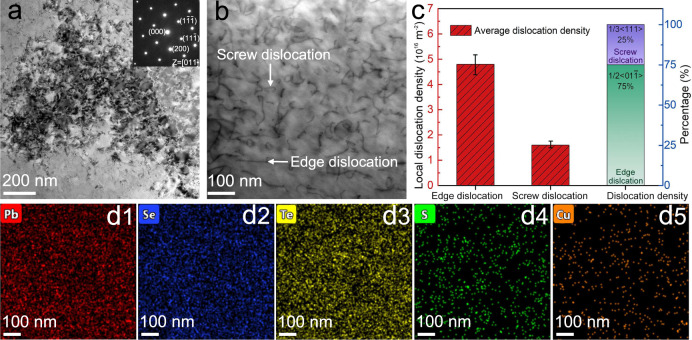
Dense dislocations in Pb_1.02_Se_0.72_Te_0.20_S_0.08_−0.3%Cu. **a** Low-magnification TEM image, the insert is the electron diffraction pattern along [011]. **b** Medium-magnification STEM ABF image. **c** Estimation of dislocation density and fraction. Error bars are ±10%. **d1**–**d5** EDS mapping of Pb, Se, Te, S, and Cu in **b**.

To directly see the dislocation in matrix, the scanning transmission electron microscopy (STEM) is carried out. Large-scale dislocation networks form in Pb_1.02_Se_0.72_Te_0.20_S_0.08_−0.3%Cu from low-magnification TEM image (Fig. 4a), and the matrix still preserves cubic PbSe-based phase from electron diffraction pattern inset. In medium-magnification STEM ABF image (Fig. 4b), dense dislocations can be clearly observed and both edge and screw dislocations exist in Pb_1.02_Se_0.72_Te_0.20_S_0.08_−0.3%Cu. By counting the local dislocation, the estimated total dislocation density is around 6.4 × 10^16 ^m^−2^, which is consistent with the modified Williamson-Hall results above. The statistical results further reveal 75% edge dislocation and 25% screw dislocation in Pb_1.02_Se_0.72_Te_0.20_S_0.08_−0.3%Cu (Fig. 4c). Although dense dislocations form in matrix, the energy dispersive spectroscopy (EDS) mapping shows all the elements are homogeneously distributed in medium-magnification area.

**Fig. 5:**
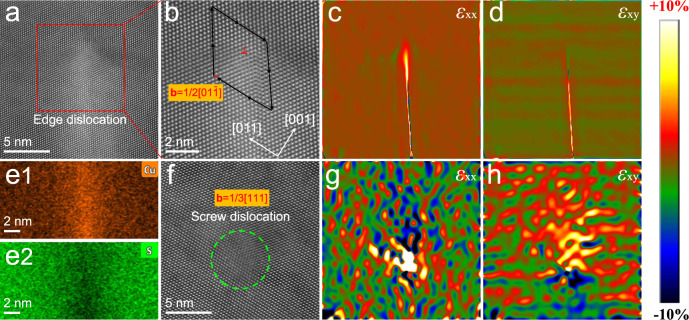
The feature of the edge dislocation and screw dislocation in Pb_1.02_Se_0.72_Te_0.20_S_0.08_−0.3%Cu. **a** HAADF-STEM image of one edge dislocation. **b** Burgers vector of the corresponding region of **a**. **c**, **d** GPA strain analysis of **a**. **e1**, **e2** EDS mapping of Cu and S at edge dislocation. **f** HRTEM HADDF image of one screw dislocation. **g**, **h** GPA strain analysis of **f**.

Furthermore, HADDF-STEM image and strain mapping of Pb_1.02_Se_0.72_Te_0.20_S_0.08_−0.3%Cu display more details on the dislocation and the strain distribution (Fig. 5). One clear edge dislocation can be witnessed (Fig. 5a), and the corresponding Burgers vector **b** = 1/2[01$\overline{1}$] is defined by Burgers circuit (Fig. 5b). The geometric phase analysis (GPA) results (Fig. 5c, d) demonstrate that edge dislocation could cause obvious linear strain field in lattice along both strain tensor *ε*_xx_ and *ε*_yy_. The EDS mapping results (Fig. 5e and Supplementary Fig. 7) show that there is Cu-rich area around the edge dislocation, which indicates the role of Cu interstitials to promote dislocation formation. Another screw dislocation is presented with a Burgers vector **b** = 1/3[111] (Fig. 5f). Moreover, the GPA results of this screw dislocation also unclose strong strain fields (Fig. 5g, h). These strain fields induced by both edge and screw dislocations can reinforce the phonon scattering to largely reduce the lattice thermal conductivity. Notably, the screw dislocation could play a stronger role to scatter phonon transport than edge dislocation due to its larger-scale strain field. The different roles between edge and screw dislocations could provide new and detailed understandings of dislocation to scatter phonon, and can also provide reference for importing dislocation in thermoelectric materials.

### Electrical transport properties in Pb_1.02_Se_0.72_Te_0.20_S_0.08_-*x*%Cu (*x* = 0–0.40)

**Fig. 6:**
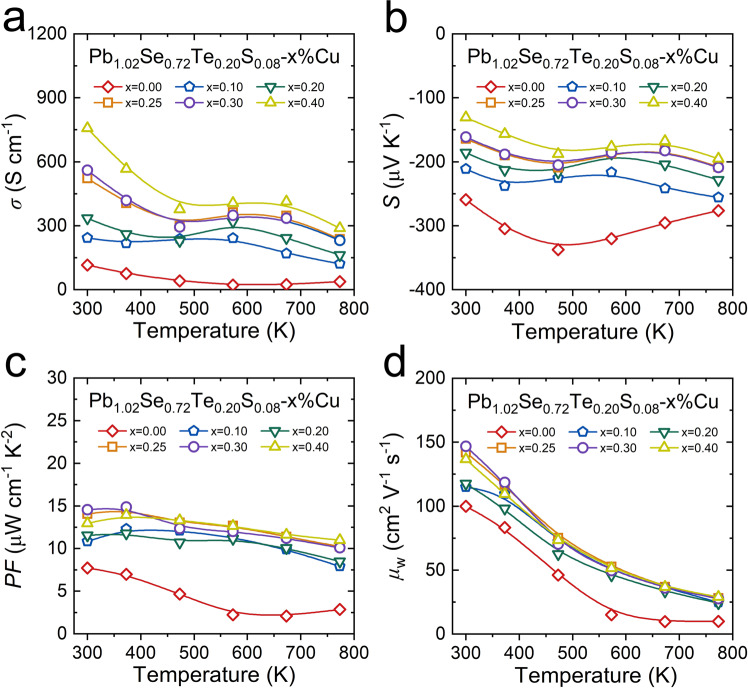
Temperature-dependent electrical transport properties in Pb_1.02_Se_0.72_Te_0.20_S_0.08_*-x*%Cu. **a** Electrical conductivity. **b** Seebeck coefficient. **c** Power factor. **d** Weighted carrier mobility (*μ*_W_).

Although dense dislocations are imported in Pb_1.02_Se_0.72_Te_0.20_S_0.08_-*x*%Cu (*x* = 0–0.40), the matrix can also maintain high electrical transport performance. With increasing Cu interstitial content in Pb_1.02_Se_0.72_Te_0.20_S_0.08_-*x*%Cu (*x* = 0–0.40), the electrical conductivity continues to rise because Cu interstitial doping can release extra free electrons into matrix (Fig. 6a). The enhanced electrical conductivity is consistent with the lowered absolute value of Seebeck coefficient (Fig. 6b). Moreover, the down-and-up tendency in temperature-dependent electrical conductivity and Seebeck coefficient indicates the dynamic doping behavior of Cu interstitials in lead chalcogenides as proved in our previous works^20,42,43^. Benefiting from the Cu interstitial doping, the power factor in Pb_1.02_Se_0.72_Te_0.20_S_0.08_-*x*%Cu (*x* = 0–0.40) is obviously enhanced in the whole-temperature range. The maximum power factor is enhanced by double, from 7.73 μW cm^−1^ K^2^ in Pb_1.02_Se_0.72_Te_0.20_S_0.08_ to 14.6 μW cm^−1^ K^2^ in Pb_1.02_Se_0.72_Te_0.20_S_0.08_−0.3%Cu (Fig. 6c). Here, the weighted carrier mobility (*μ*_W_) is introduced to evaluate the contribution of Cu atom to electrical transport properties. The parameter *μ*_W_ can be calculated with measured electrical conductivity and Seebeck coefficient by the following relationships^44^:

3 $${\mu }_{{{{{{\rm{W}}}}}}}=\frac{3\sigma }{8\pi e{F}_{0}(\eta )}{\left(\frac{{h}^{2}}{2{m}_{{{{{{\rm{e}}}}}}}{k}_{{{{{{\rm{B}}}}}}}T}\right)}^{3/2}$$

4 $${F}_{{{{{{\rm{n}}}}}}}(\eta )={\int }_{0}^{\infty }\frac{{x}^{{{{{{\rm{n}}}}}}}}{1+{e}^{x-\eta }}dx$$

5 $$S= \pm \frac{{k}_{{{{{{\rm{B}}}}}}}}{e}\left(\frac{(r+5/2){F}_{r+3/2}(\eta )}{(r+3/2){F}_{r+1/2}(\eta )}-\eta \right)$$

where *e*, *h*, *m*_e_, and *k*_B_ donate the unite charge, Planck constant, electron mass, and the Boltzmann constant, respectively. *F*_n_(*η*) is the Fermi integral, *η* is reduced Fermi level, and *r* is the scattering factor and equals −1/2 when the acoustic scattering mechanism dominates. The temperature-dependent *μ*_W_ of Pb_1.02_Se_0.72_Te_0.20_S_0.08_-*x*%Cu (*x* = 0–0.40) (Fig. 6d) shows that Cu interstitial doping can well promote *μ*_W_ and a maximum value of 146.90 cm^2^ V^−1^ s^−1^ is obtained in Pb_1.02_Se_0.72_Te_0.20_S_0.08_−0.3%Cu at room temperature, which is the origin of the enhanced power factor.

### Thermal transport properties and *ZT* value in Pb_1.02_Se_0.72_Te_0.20_S_0.08_-*x*%Cu (*x* = 0–0.40)

**Fig. 7:**
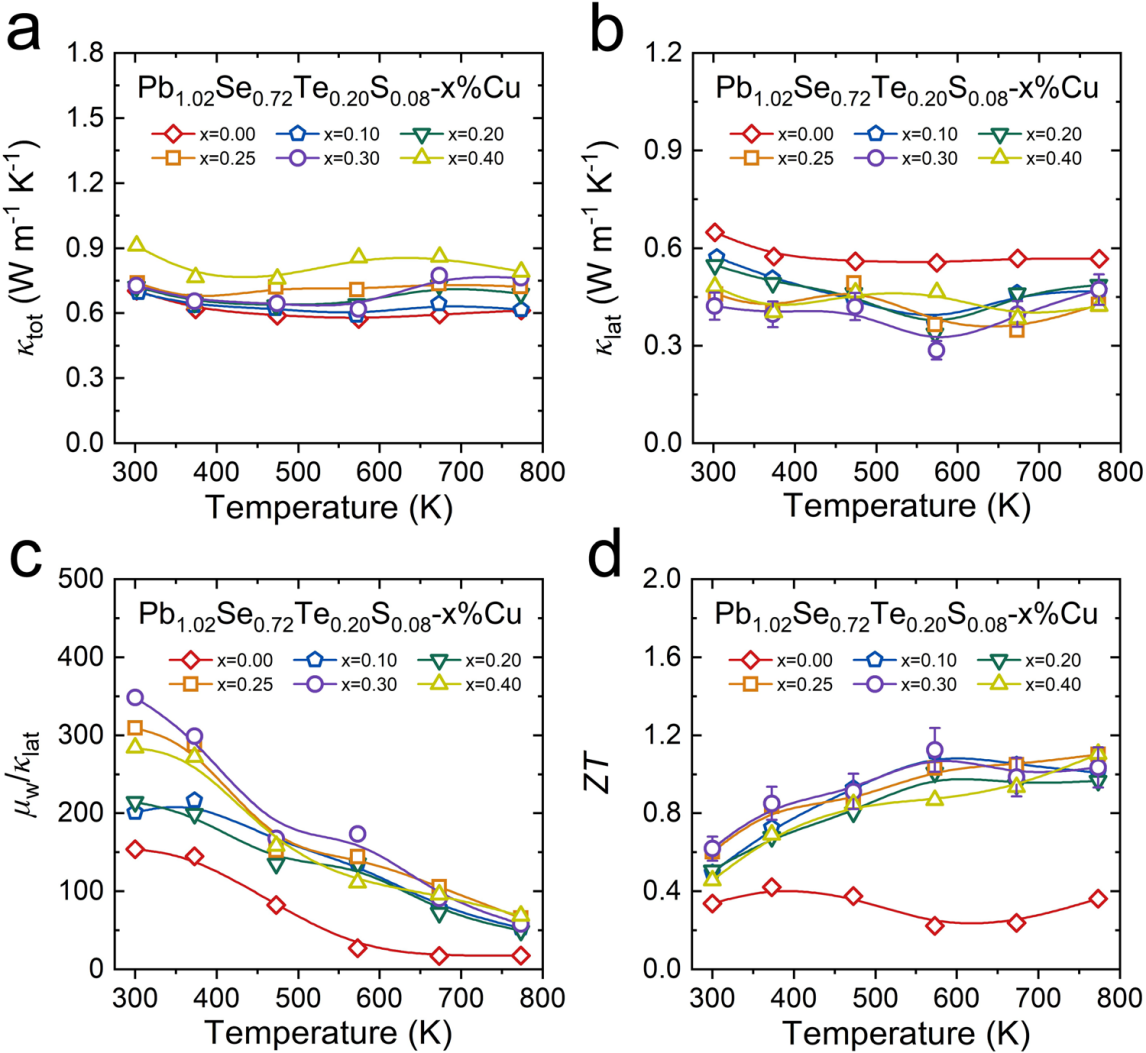
Temperature-dependent thermal conductivity and *ZT* values in Pb_1.02_Se_0.72_Te_0.20_S_0.08_-*x*%Cu. **a** Total thermal conductivity. **b** Lattice thermal conductivity. Error bars are ±10%. **c** Ratio of weighted carrier mobility to lattice thermal conductivity. **d** *ZT* values. Error bars are ±10%.

Owning to the increased electrical conductivity, the total thermal conductivity in Pb_1.02_Se_0.72_Te_0.20_S_0.08_-*x*%Cu (*x* = 0–0.40) undergoes an increasing tendency after Cu interstitial doping (Fig. 7a). With electronic thermal conductivity (Supplementary Fig. 8), the lattice thermal conductivity in Pb_1.02_Se_0.72_Te_0.20_S_0.08_-*x*%Cu (*x* = 0–0.40) is calculated out (Fig. 7b). Moreover, benefiting from the dense dislocations in Pb_1.02_Se_0.72_Te_0.20_S_0.08_-*x*%Cu (*x* = 0–0.40) as discussed above, the lattice thermal conductivity can be further suppressed with Cu interstitial doping. To evaluate the synergistic role of Cu interstitial doping in Pb_1.02_Se_0.72_Te_0.20_S_0.08_-*x*%Cu (*x* = 0–0.40), the temperature-dependent ratio of weighted carrier mobility to lattice thermal conductivity (*µ*_W_/*κ*_lat_) is calculated out (Fig. 7c). The temperature-dependent *µ*_W_/*κ*_lat_ values show decreasing tendency with rising temperature, but obtain large enhancement after Cu interstitial doping. Finally, the *ZT* values are distinctly enhanced in Pb_1.02_Se_0.72_Te_0.20_S_0.08_-*x*%Cu (*x* = 0–0.40) (Fig. 7d). Notably, the room-temperature *ZT* value of Pb_1.02_Se_0.72_Te_0.20_S_0.08_−0.3%Cu can reach at 0.62, indicating potential application of PbSe-based thermoelectric near room temperature. Additionally, the cycle test results (Supplementary Fig. 9) further demonstrate a good repeatability and high reliability of high-performance Pb_1.02_Se_0.72_Te_0.20_S_0.08_−0.3%Cu.

**Fig. 8:**
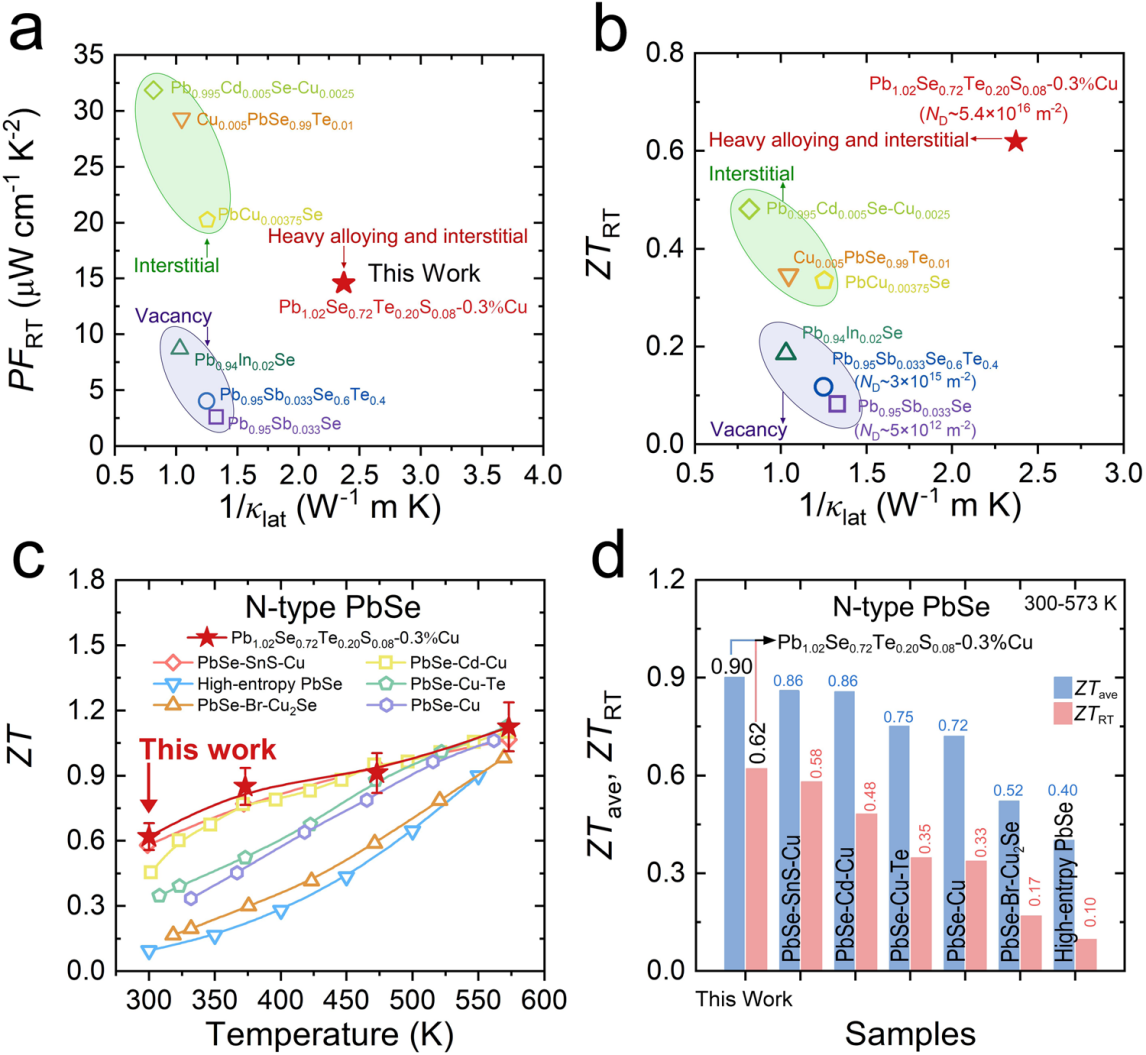
Comparisons of thermoelectric transport performance in *n*-type PbSe-based thermoelectric materials. **a** *PF* value as a function of 1/*κ*_lat_ at room temperature. **b** *ZT* value as a function of 1/*κ*_lat_ at room temperature. **c** Temperature-dependent *ZT* value. Error bars are ± 10%. **d** *ZT*_ave_ value at 300–573 K and *ZT* value at room temperature.

The room-temperature thermoelectric properties between Pb_1.02_Se_0.72_Te_0.20_S_0.08_−0.3%Cu in this work and other *n*-type PbSe-based samples with dislocation are compared (Fig. 8a, b). It is found that all the samples present low lattice thermal conductivity, proving the positive role of dislocation to scatter phonon. Interestingly, the Cu interstitial-induced dislocation in *n*-type PbSe can result in better electrical transport properties than that in samples with Pb vacancy-induced dislocation. This might be closely related to the local carrier types (electron or hole) near different dislocations. Specifically, the interstitial-induced dislocation is dominated by donor electron that is beneficial to *n*-type sample, but the cation vacancy-induced dislocation contains local hole carrier, which could cause energy barrier for electrical transport in *n*-type thermoelectric materials. Owning to much higher dislocation density in Pb_1.02_Se_0.72_Te_0.20_S_0.08_−0.3%Cu, this work can obtain very low lattice thermal conductivity and simultaneously maintain relatively high power factor, which is conducive to the improvement of *ZT* value at room temperature (Fig. 8c) and enhance the *ZT*_ave_ value in 300–573 K. Compared with other state-of-the-art *n*-type PbSe, Pb_1.02_Se_0.72_Te_0.20_S_0.08_−0.3%Cu presents a superior *ZT* value at 300–573 K (Fig. 8d). Moreover, its *ZT*_RT_ and *ZT*_ave_ value at 300–573 K can reach up to 0.62 and 0.90, respectively, outperforming other reported high-performance *n*-type PbSe-based thermoelectric materials, such as *ZT*_RT_ ~ 0.58 and *ZT*_ave_ ~ 0.86 in PbSe-SnS-Cu^20^, *ZT*_RT_ ~ 0.48 and *ZT*_ave_ ~ 0.86 in PbSe-Cd-Cu^30^, *ZT*_RT_ ~ 0.35 and *ZT*_ave_ ~ 0.75 in PbSe-Cu-Te^29^, *ZT*_RT_ ~ 0.33 and *ZT*_ave_ ~ 0.72 in PbSe-Cu^22^, *ZT*_RT_ ~ 0.17 and *ZT*_ave_ ~ 0.52 in PbSe-Br-Cu_2_Se^19^, *ZT*_RT_ ~ 0.10 and *ZT*_ave_ ~ 0.40 in high-entropy PbSe^32^. More importantly, this high *ZT*_ave_ value in Pb_1.02_Se_0.72_Te_0.20_S_0.08_−0.3%Cu is comparable with the thermoelectric performance in typical *n*-type Bi_2_Te_3_-based materials, including commercial Bi_2_Te_2.5_Se_0.5_^45^, Bi_2_Te_2.7_Se_0.3_ + 1 wt%Bi_2_S_3_^46^, and Bi_2_Te_3 _+ 1 wt%Ru^47^ (Supplementary Fig. 10). The present high near-room-temperature thermoelectric performance indicates that Pb_1.02_Se_0.72_Te_0.20_S_0.08_−0.3%Cu has great potential for thermoelectric cooling applications at low-medium temperatures.

## Discussion

In summary, high-density dislocation is realized in Pb_1.02_Se_0.72_Te_0.20_S_0.08_−0.3%Cu through heavy alloying and interstitial doping. Such dense dislocations can form dislocation networks and largely reduce the lattice thermal conductivity in the whole-temperature range. The room-temperature and minimal lattice thermal conductivity can be suppressed to 0.42 W m^−1^ K^−1^ and 0.29 W m^−1^ K^−1^ in Pb_1.02_Se_0.72_Te_0.20_S_0.08_−0.3%Cu. Additionally, Cu interstitials not only promotes dislocation formation to intensify phonon scattering but also works as donor dopant to optimize carrier transport properties. The resultant reduced thermal conductivity and preserved electrical transport properties finally contribute to a high *ZT*_ave_ of 0.96 in *n*-type Pb_1.02_Se_0.72_Te_0.20_S_0.08_−0.3%Cu at low-medium temperatures (300–773 K), which mainly originates from its enhanced performance at low temperature range (300–573 K). The work provides a new approach to enhance the thermoelectric performance in lead chalcogenides at low-medium temperatures, and the strategy of heavy alloying and interstitial doping to produce dense dislocations can be also extended to other thermoelectric semiconductors.

## Methods

### Synthesis

High-purity raw materials, Pb bulk (99.999%), Se particles (99.999%), Te bulk (99.999%), S powders (99.99%), and Cu wires (99.99%) with stoichiometric composition were weighted and flame-sealed in silica tubes at a residual pressure below ~10^−4^ Torr, slowly heated to 1323 K in 12 h and kept at this temperature for 6 h followed by furnace cooling to room temperature. The obtained ingots were ground into powders and densified by hot-pressing furnace (OTF-1700X-RHP4) at 773 K for 40 min in a Ф 15 mm cylindrical die under an axial compressive stress of 50 MPa in vacuum, resulting in highly densified disk-shaped samples. Finally, the disk-shaped samples were annealed at 773 K for 6 h.

### Structural characterization

The phase identification was characterized through powder X-ray diffraction with Cu Kα (*λ* = 1.5418 Å) radiation in a reflection geometry operating at 40 kV and 40 mA. The lattice parameters were calculated and refined by using the software package, which named “Materials Analysis using Diffraction (MAUD)”. The transmission electron microscopy (TEM) and high-resolution TEM (HRTEM) image as well as the scanning transmission electron microscopy (AC-STEM) HAADF image were conducted on a spherical aberration corrected Transmission Electron Microscope (Thermalfisher Titan Themis Z). TEM samples are prepared by focused ion beam (FIB) method on Thermalfisher Scios 2.

### Thermoelectric transport properties measurements

The obtained highly densified hot-pressing (HP) processed disk-shaped samples were cut into bars with dimensions around ~12 × ~4  × ~4 mm to measure electrical conductivity (*σ*) and Seebeck coefficient (*S*) from 300 to 773 K by using a CTA equipment (Cryoall, China) under a low-pressure helium atmosphere. The HP disk-shaped samples were cut and polished into a square with sizes of ~8 × ~8 mm and ~1.5 mm thickness for thermal diffusivity (*D*) measurements. The samples were coated with a thin-graphite layer to minimize errors from the emissivity method in LFA-467 (Netzsch, Germany) and CLA-1000 (Cryoall, China) instruments. The sample density (*ρ*) was determined using the dimensions and mass of the samples. The specific heat capacity (*C*_p_) was calculated by the Debye model. The thermal diffusivity data was analyzed using a Cowan model with pulse correction.

## Supplementary information

Supplementary Information

## Data Availability

The authors declare that the data supporting the findings of this study are available on reasonable request.
