# Supplementary material for: Dense dislocations enable high-performance PbSe thermoelectric at low-medium temperatures
Source: Nat Commun. 2022 Oct 28;13:6449. doi: 10.1038/s41467-022-34227-3 (PMC9616947; doi:10.1038/s41467-022-34227-3)
Supplement: Supplementary file 1 — Supplementary Information [file 41467_2022_34227_MOESM1_ESM.pdf]

Supplementary Information

Dense dislocations enable high-performance PbSe thermoelectric at low-medium temperatures

Liqing Xu,[1] Yu Xiao,[1,*] Sining Wang,[2] Bo Cui,[3,*] Di Wu,[4] Xiangdong Ding,[1] and Li-Dong Zhao [2,*]

[1]State Key Laboratory for Mechanical Behavior of Materials, Xi'an Jiaotong University, Xi'an 710049, China.

[2]School of Materials Science and Engineering, Beihang University, Beiijng 100191, China.

[3]Institute of Nuclear Physics and Chemistry, China Academy of Engineering Physics, Mianyang, 621900, China.

[4]School of Materials Science and Engineering, Shaanxi Normal University, Xi'an 710049, China.

Corresponding author: xiao_yu@xjtu.edu.cn (Y. Xiao), cuibo13@163.com (B. Cui), zhaolidong@buaa.edu.cn (L.-D. Zhao).

Supplementary Methods

Lorenz number calculation. The Lorenz number (L) is used to evaluate the electronic thermal conductivity (κ_{ele}) with a Wiedemann-Franz law of $\kappa_{ele}=L\sigma T$, where the σ is the electrical conductivity, T denotes working temperature. The Lorenz number can be obtained by fitting the Seebeck coefficient to the reduced chemical potential with following equations:

$$L=\left(\frac{k_B}{e}\right)^2\left(\frac{(r+7/2)F_{r+5/2}(\eta)}{(r+3/2)F_{r+1/2}(\eta)}-\left[\frac{(r+5/2)F_{r+3/2}(\eta)}{(r+3/2)F_{r+1/2}(\eta)}\right]^2\right) \quad \text{(S1)}$$

$$S=\pm\frac{k_B}{e}\left(\frac{(r+5/2)F_{r+2/3}(\eta)}{(r+3/2)F_{r+1/2}(\eta)}-\eta\right) \quad \text{(S2)}$$

where the $F_n(\eta)$ is the n-th order Fermi integral:

$$F_n(\eta)=\int_0^\infty \frac{x^n}{1+e^{x-\eta}}dx \quad \text{(S3)}$$

$$\eta=\frac{E_f}{k_B T} \quad \text{(S4)}$$

where k_B is the Boltzmann constant, e is the electron charge and E_f denotes the Fermi level, r is the scattering factor, and the acoustic phonon scattering has been assumed as the main carrier scattering mechanism with r=-1/2.

Heat capacity calculation. The heat capacity in this work was theoretically evaluated by Debye model. The Debye model considers the individual contributions of phonons and effects of thermal expansion of lattice. The temperature-dependent total heat capacity ($C_{p,\,tot}$) can be obtained with following relationships:

$$C_{p,\,tot}=C_{p,\,ph}(T)+C_{p,\,D}(T) \quad \text{(S5)}$$

where $C_{p,\,ph}$ and $C_{p,\,D}$ denote heat capacity originated from phonon and lattice dilation, respectively. The phonon heat capacity $C_{p,\,ph}$ can be calculated by:

$$C_{p,\,ph}(T/\Theta_D)=9R\left(\frac{T}{\Theta_D}\right)^3\int_0^{\Theta_D/T}\frac{x^4e^x}{(e^x-1)^2}dx \quad \text{(S6)}$$

where Θ_D is Debye temperature, R is molar gas constant, $x=\hbar\omega/k_B$T, in which $\hbar$ and

denote reduced Planck constant and phonon vibration frequency, respectively.

The effects of lattice dilation on heat capacity $C_{\mathrm{p,\,D}}$ can be obtained from:

$$C_{\mathrm{p,\,D}}(T) = C_{\mathrm{ele,\,D}}(T) + C_{\mathrm{ph,\,D}}(T) = \frac{9BT\alpha^2}{10^6\rho} \tag{S7}$$

where B is the isothermal bulk modulus, α is the linear coefficient of thermal expansion, and ρ is sample density. Notably, the electron heat capacity is not taken into account due to its negligible effects on lattice dilation compared with phonon.

Callaway model to predict lattice thermal conductivity. Callaway model shows the ratio of the conductivities of material containing defects to that of pure material[1-3]:

$$\frac{\kappa_{\mathrm{lat}}}{\kappa_{\mathrm{lat},p}} = \frac{\tan^{-1}(u)}{u} \tag{S8}$$

in which κ_{lat} and $\kappa_{\mathrm{lat},p}$ are the lattice thermal conductivities of the defected and parent materials, respectively, and $\kappa_{\mathrm{lat},p}$=1.68 W m^{-1} K^{-1} in this work. u is defined as[2, 4]:

$$u = \left(\frac{\pi^2 \Theta_{\mathrm{D}} \Omega}{h v_{\mathrm{a}}^2} \kappa_{\mathrm{lat},p} \Gamma\right)^{1/2} \tag{S9}$$

where Ω and h are the average atom volume and Planck constant, respectively. The Debye temperature (Θ_{D}), average sound velocity (v_{a}) and can be written as[5]:

$$\Theta_{\mathrm{D}} = \frac{h}{k_{\mathrm{B}}}\left(\frac{3}{4\pi\Omega}\right)^{1/3} v_{\mathrm{a}} \tag{S10}$$

$$v_{\mathrm{a}} = \left[\frac{1}{3}\left(\frac{1}{v_{\mathrm{l}}^3} + \frac{1}{v_{\mathrm{s}}^3}\right)\right]^{-1/3} \tag{S11}$$

where k_{B}, v_{l} and v_{s} are Boltzmann constant, longitudinal and shear sound velocities, respectively. In this work, there is no change on the sites of Pb after Q (Te or S) substituting Se sites, namely the imperfection scattering parameter Γ_{Pb}=0, Thus, the $\Gamma_{\mathrm{Pb_{1.02}Se_{1\text{-}x}Q_x}}$ is defined as[6]:

$$\Gamma_{\mathrm{Pb_{1.02}Se_{1\text{-}x}Q_x}} = \frac{1}{2}\left(\frac{M_{\mathrm{(Se,\,Q)}}}{\overline{M}}\right)^2 \Gamma_{\mathrm{(Se,\,Q)}} \tag{S12}$$

$$\overline{M} = \frac{1}{2}(M_{\mathrm{Se}} + M_{\mathrm{Q}}) \tag{S13}$$

where M is molar mass. Meanwhile, Γ is a weighted sum of the mass fluctuation (Γ_M) and strain field fluctuation (Γ_S), can be written as[3, 7]:

$$\Gamma_{(\mathrm{Se},\,\mathrm{Q})} = \Gamma_{M(\mathrm{Se},\,\mathrm{Q})} + \varepsilon\Gamma_{S(\mathrm{Se},\,\mathrm{Q})} \tag{S14}$$

in which ε is a phenomenological adjustable parameter related to the Poisson ratio (v_p) and Grüneisen parameter (γ). Moreover, they can be expressed by[8, 9]:

$$\varepsilon = \frac{2}{9}\left[\left(G + 6.4\gamma\right)\frac{1+v_\mathrm{p}}{1-v_\mathrm{p}}\right]^2 \tag{S15}$$

$$\gamma = \frac{3}{2}\left(\frac{1+v_\mathrm{p}}{2-3v_\mathrm{p}}\right) \tag{S16}$$

$$v_\mathrm{p} = \frac{1-2(v_\mathrm{s}/v_\mathrm{l})^2}{2-2(v_\mathrm{s}/v_\mathrm{l})^2} \tag{S17}$$

where G is a ratio between the relative change of bulk modulus and banding length. And $\Gamma_{M(\mathrm{Se},\,\mathrm{Q})}$ and $\Gamma_{S(\mathrm{Se},\,\mathrm{Q})}$ in equation (S14) can be expended as follows:

$$\Gamma_{M(\mathrm{Se},\,\mathrm{Q})} = x(1-x)\left(\frac{\Delta M}{M_{(\mathrm{Se},\,\mathrm{Q})}}\right)^2 \tag{S18}$$

$$\Gamma_{S(\mathrm{Se},\,\mathrm{Q})} = x(1-x)\left(\frac{\Delta r}{r_{(\mathrm{Se},\,\mathrm{Q})}}\right)^2 \tag{S19}$$

where ΔM, Δr and r can be written as[7]:

$$M_{(\mathrm{Se},\,\mathrm{Q})} = (1-x)M_{\mathrm{Se}} + xM_{\mathrm{Q}} \tag{S20}$$

$$\Delta M = M_{\mathrm{Se}} - M_{\mathrm{Q}} \tag{S21}$$

$$r_{(\mathrm{Se},\,\mathrm{Q})} = (1-x)r_{\mathrm{Se}} + xr_{\mathrm{Q}} \tag{S22}$$

$$\Delta r = r_{\mathrm{Se}} - r_{\mathrm{Q}} \tag{S23}$$

Then we can obtain:

$$\Gamma_{\mathrm{Pb_{1.02}Se_{1\text{-}x}Q_x}} = \frac{1}{2}x\left(1-x\right)\left(\frac{M_{(\mathrm{Se},\,\mathrm{Q})}}{\overline{M}}\right)^2\left[\left(\frac{\Delta M}{M_{(\mathrm{Se},\,\mathrm{Q})}}\right)^2 + \varepsilon\left(\frac{\Delta r}{r_{(\mathrm{Se},\,\mathrm{Q})}}\right)^2\right] \tag{S24}$$

Modified Williamson-Hall method to calculate dislocation density. In consideration of that size and strain broadening are diffraction order independent and independent, respectively, Williamson and Hall suggested that the full width at half-maximum (FWHM) of line profiles can be written as[10-12]:

$$\Delta K = 0.9/d + \Delta K^{\mathrm{d}} \tag{S25}$$

where ΔK^{d} is the strain contribution to line broadening and d is the average grain size or particle size. K=2sinθ/λ, $\Delta K = 2\cos\theta(\Delta\theta)/\lambda$, θ and λ are the diffraction angle and the wavelength of X-rays, respectively. When strain is caused by dislocations, ΔK^{d} has the following form[13-15]:

$$\Delta K^{\mathrm{d}} = A(N_{\mathrm{D}}^{*})^{1/2} + A^{'}(Q^{*})^{1/2} \tag{S26}$$

where A and A' are parameters determined by the effective outer cutoff radius of dislocations, R_e, and the auxiliary parameters R_1 and R_2, respectively. N_D^* and Q^* are the formal values of dislocation density and the correlation factors, respectively, they are related to the true values N_D and Q as[13-15]:

$$N_{\mathrm{D}}^{*} = N_{\mathrm{D}}(\pi g^2 b^2 \overline{C})/2 \tag{S27}$$

$$Q^{*} = Q(\pi g^2 b^2 \overline{C})^2/4 \tag{S28}$$

in which b is the magnitude of Burgers vector and $\overline{C}$ is the average dislocation contrast factor for a particular reflection g and g=K at the exact Bragg position. The average contrast factor is a linear function of the fourth-order invariant of the hkl indices of the different reflections[16]:

$$\overline{C} = \overline{C}_{h00}(1 - qH^2) \tag{S29}$$

where $H^2 = (h^2k^2 + h^2l^2 + k^2l^2)/(h^2 + k^2 + l^2)^2$ and $\overline{C}_{h00}$ is the average dislocation contrast factor corresponding to the h00 reflection determining by elastic modulus. Thus, equation (S25) will be written as:

$$\Delta K = 0.9/d + (\pi A^2 b^2/2)^{1/2} N_{\mathrm{D}}^{1/2}\left(K\overline{C}^{1/2}\right) \pm O(K^2\overline{C}) \tag{S30}$$

where $O = (\pi A^{'} b^2/2)Q^{1/2}$.

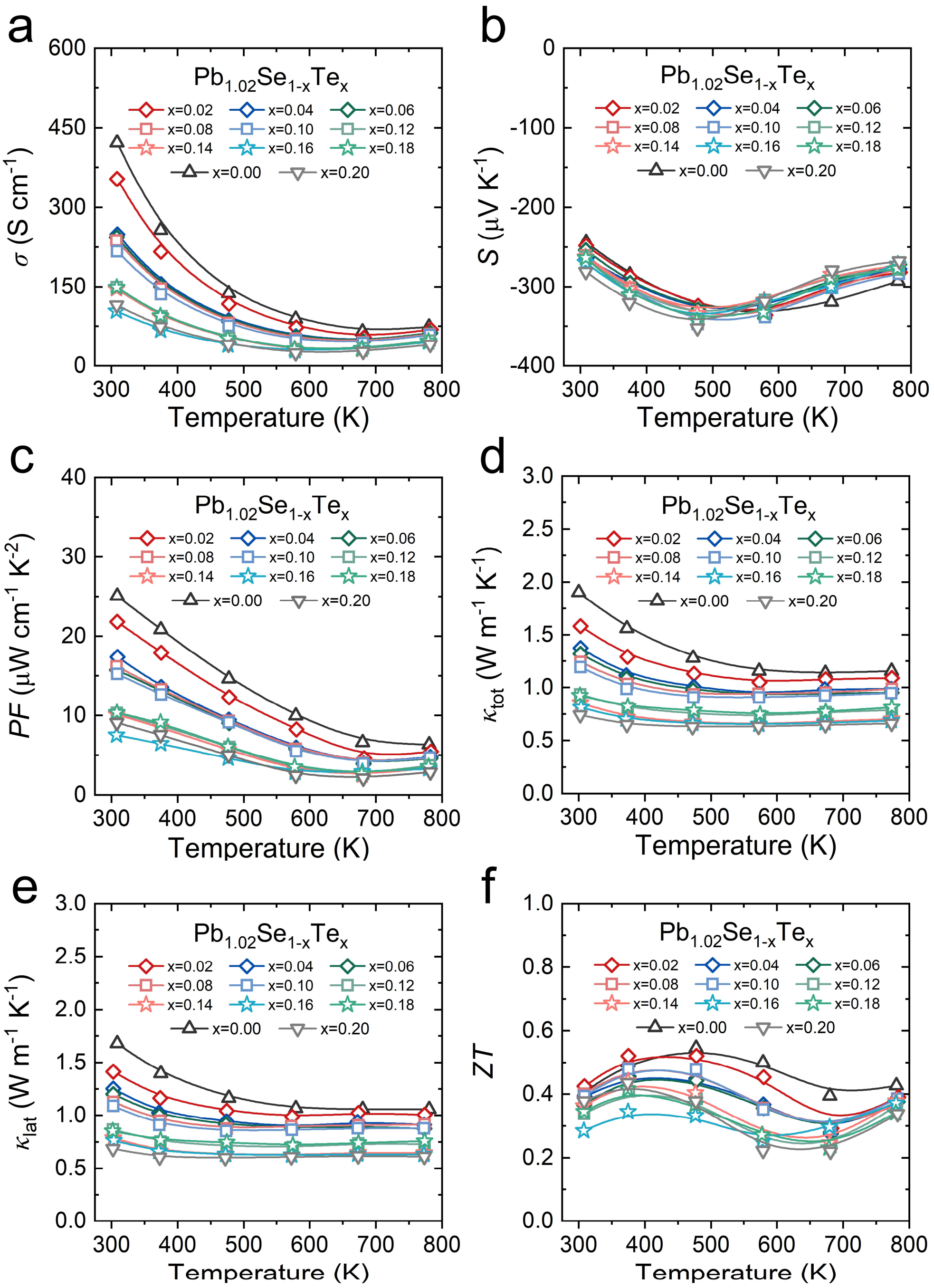

Supplementary Figure 1. Thermoelectric transport properties in $Pb_{1.02}Se_{1-x}Te_x$: (a) Electrical conductivity. (b) Seebeck coefficient. (c) Power factor. (d) Total thermal conductivity. (e) Lattice thermal conductivity. (f) *ZT* values.

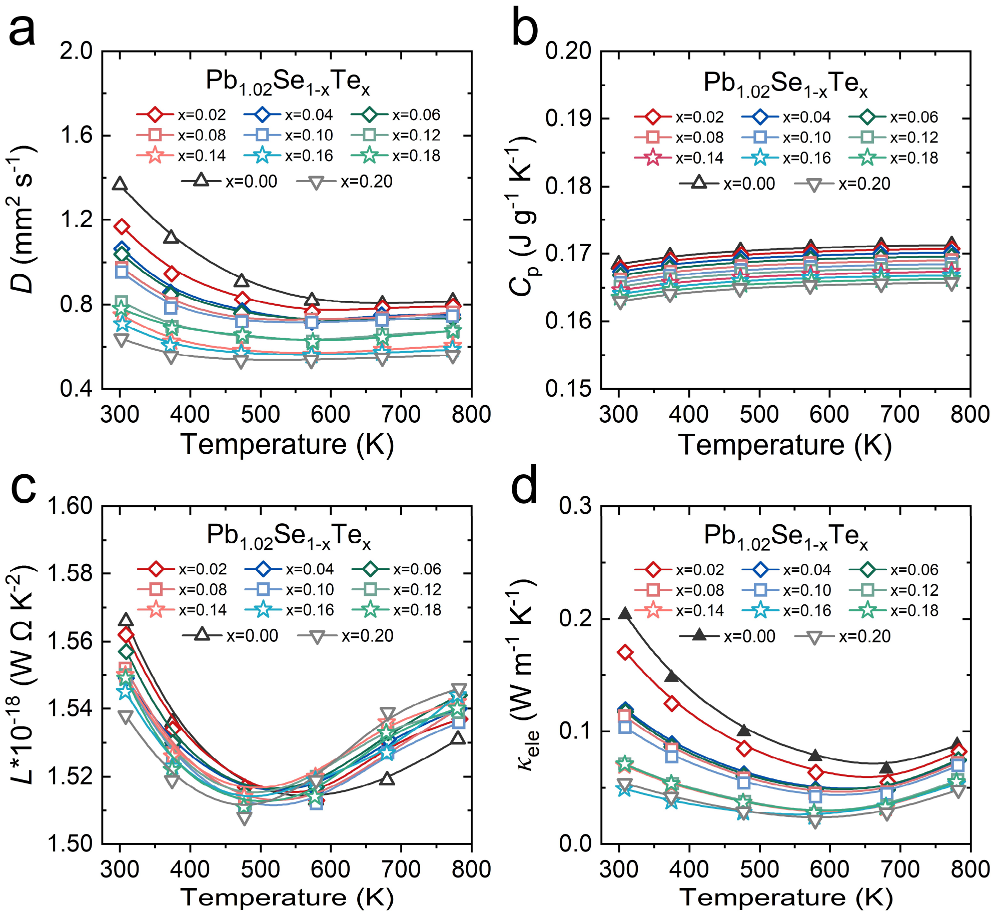

Supplementary Figure 2. Electrical transport properties for $Pb_{1.02}Se_{1-x}Te_x$: (a) Thermal diffusivity. (b) Heat capacity. (c) Lorenz number. (d) Electronic thermal conductivity.

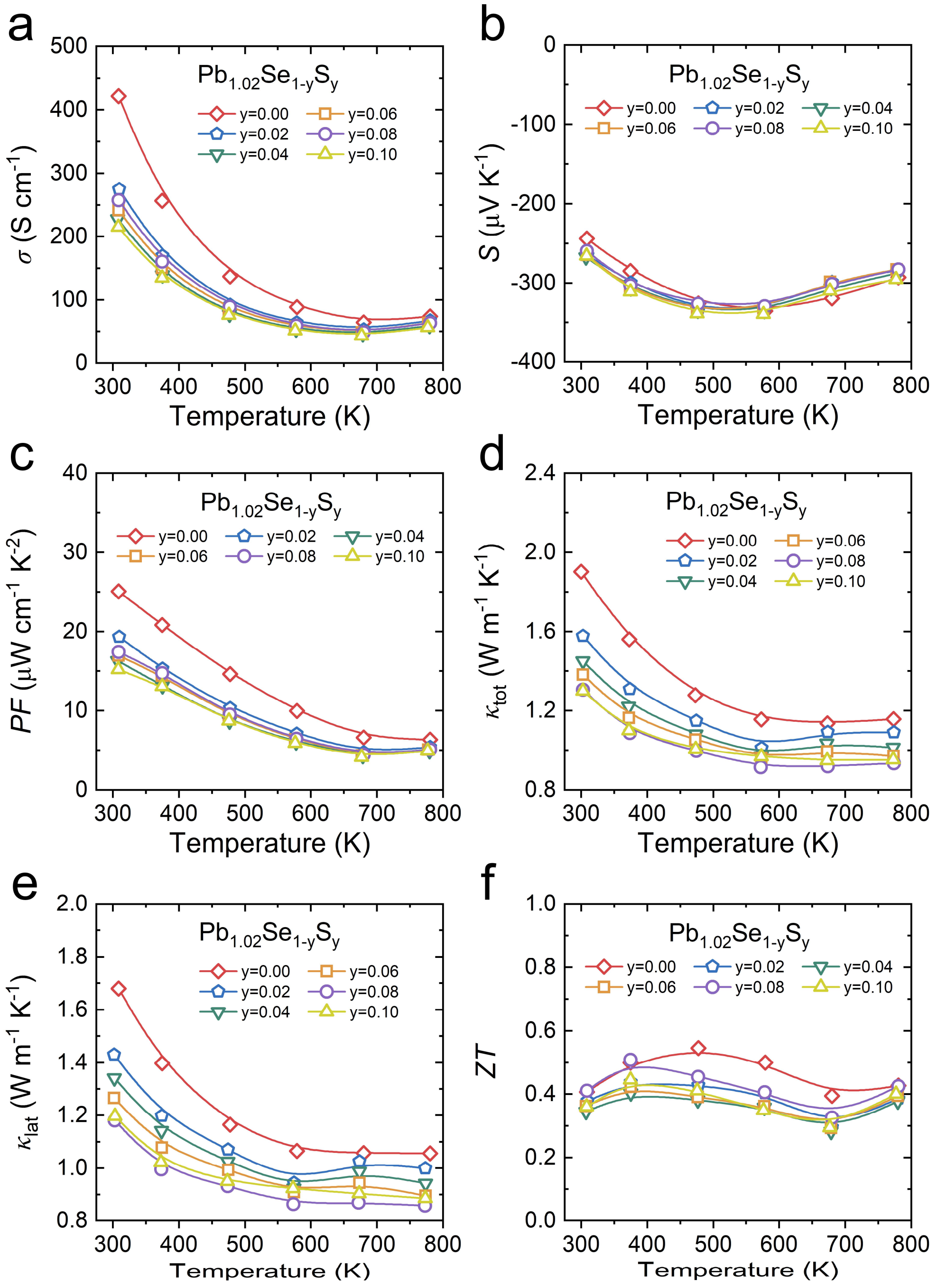

Supplementary Figure 3. Thermoelectric transport properties in $Pb_{1.02}Se_{1-y}S_y$: (a) Electrical conductivity. (b) Seebeck coefficient. (c) Power factor. (d) Total thermal conductivity. (e) Lattice thermal conductivity. (f) *ZT* values.

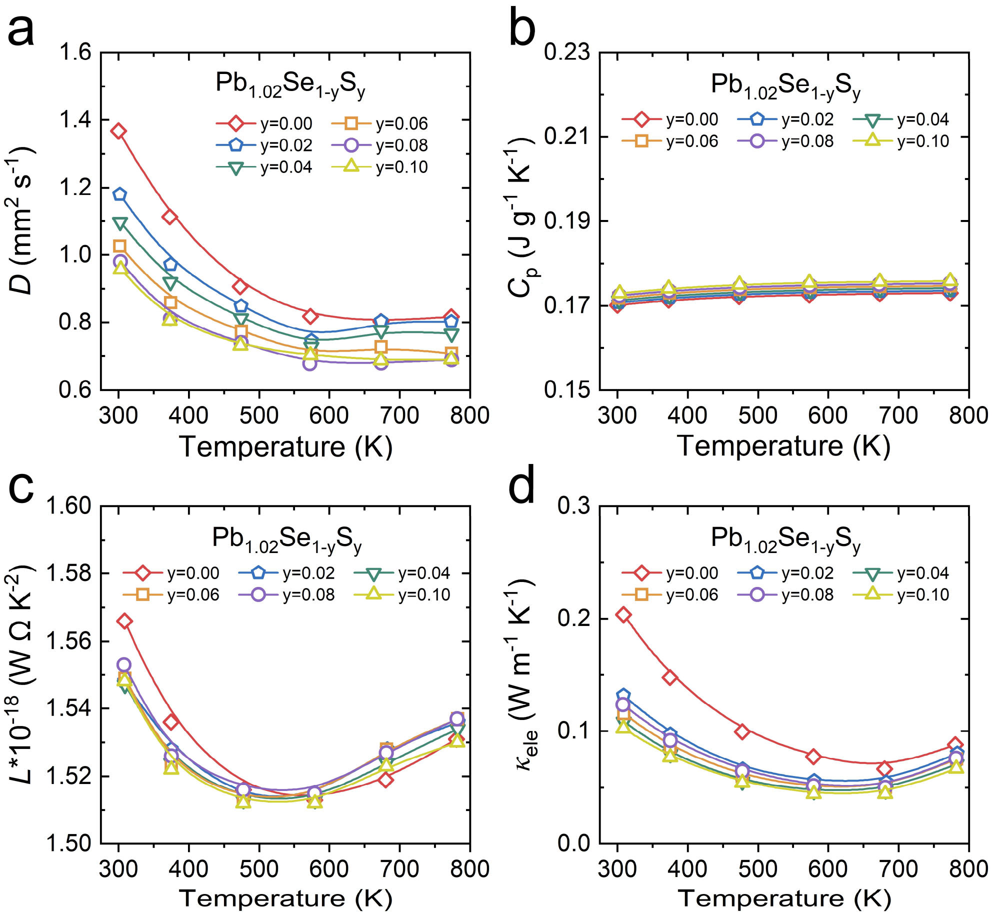

Supplementary Figure 4. Electrical transport properties for $Pb_{1.02}Se_{1-y}S_y$: (a) Thermal diffusivity. (b) Heat capacity. (c) Lorenz number. (d) Electronic thermal conductivity.

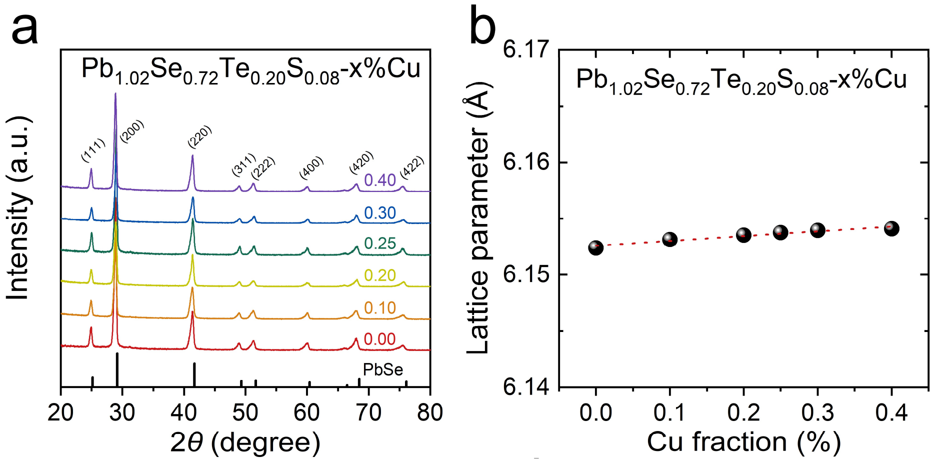

Supplementary Figure 5. Phase analysis of $Pb_{1.02}Se_{0.72}Te_{0.20}S_{0.08}$-x%Cu: (a) XRD patterns. (b) Lattice parameter as a function of Cu content.

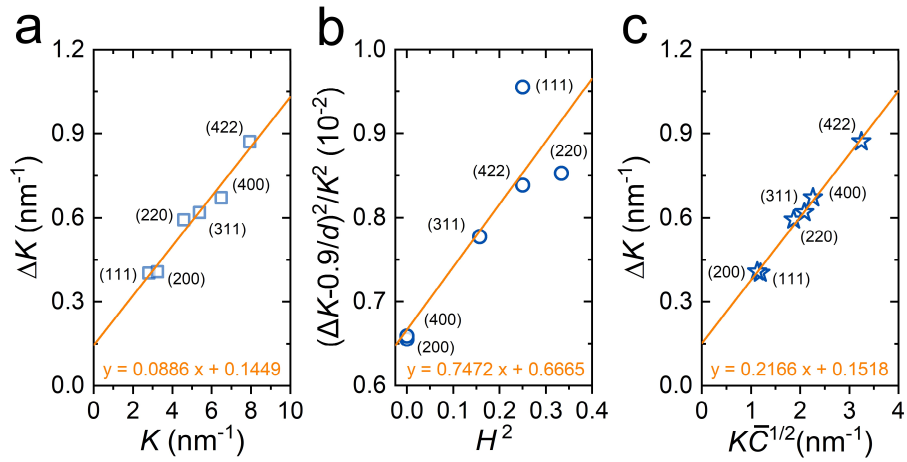

Supplementary Figure 6. Estimation of dislocation density in $Pb_{1.02}Se_{0.72}Te_{0.20}S_{0.08}$ by MWH method: (a) Plot of $\Delta K \sim K$. (b) Plot of $(\Delta K\text{-}0.9/\mathrm{d})/K^2 \sim H^2$. (c) Plot of $\Delta K \sim \overline{C}^{\,1/2}$.

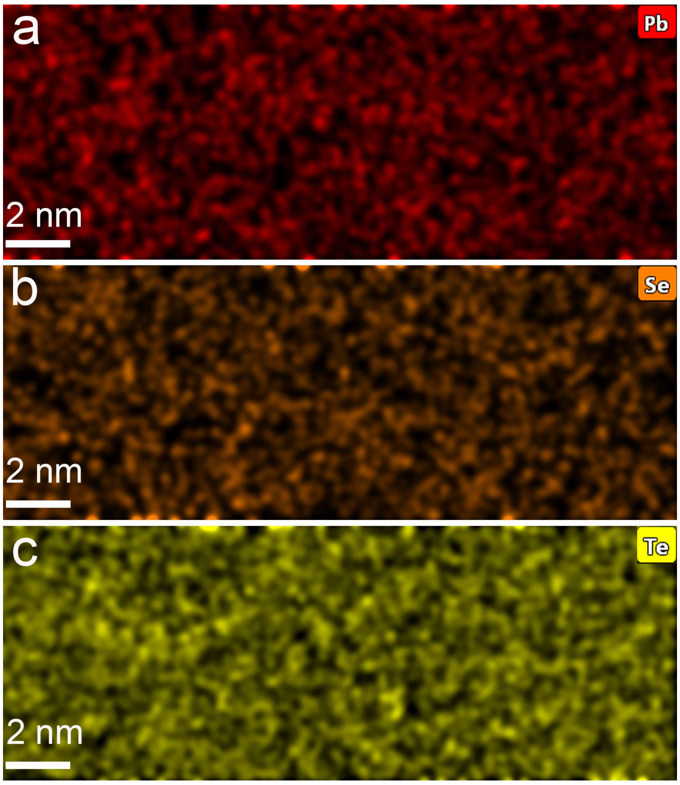

Supplementary Figure 7. EDS mapping of Pb (a), Te (b) and Se (c) at edge dislocation in $Pb_{1.02}Se_{0.72}Te_{0.20}S_{0.08}$-0.3%Cu.

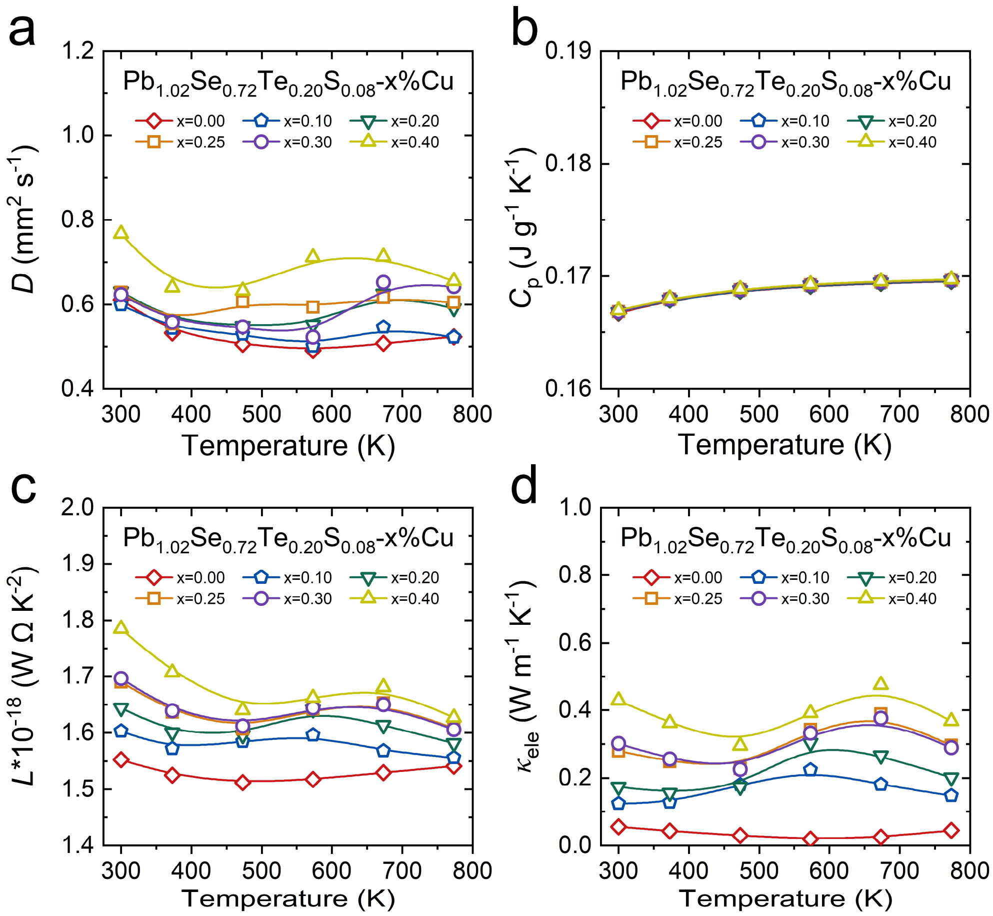

Supplementary Figure 8. Thermoelectric properties for $Pb_{1.02}Se_{0.72}Te_{0.20}S_{0.08}$-x%Cu: (a) Thermal diffusivity. (b) Heat capacity. (c) Lorenz number. (d) Electronic thermal conductivity.

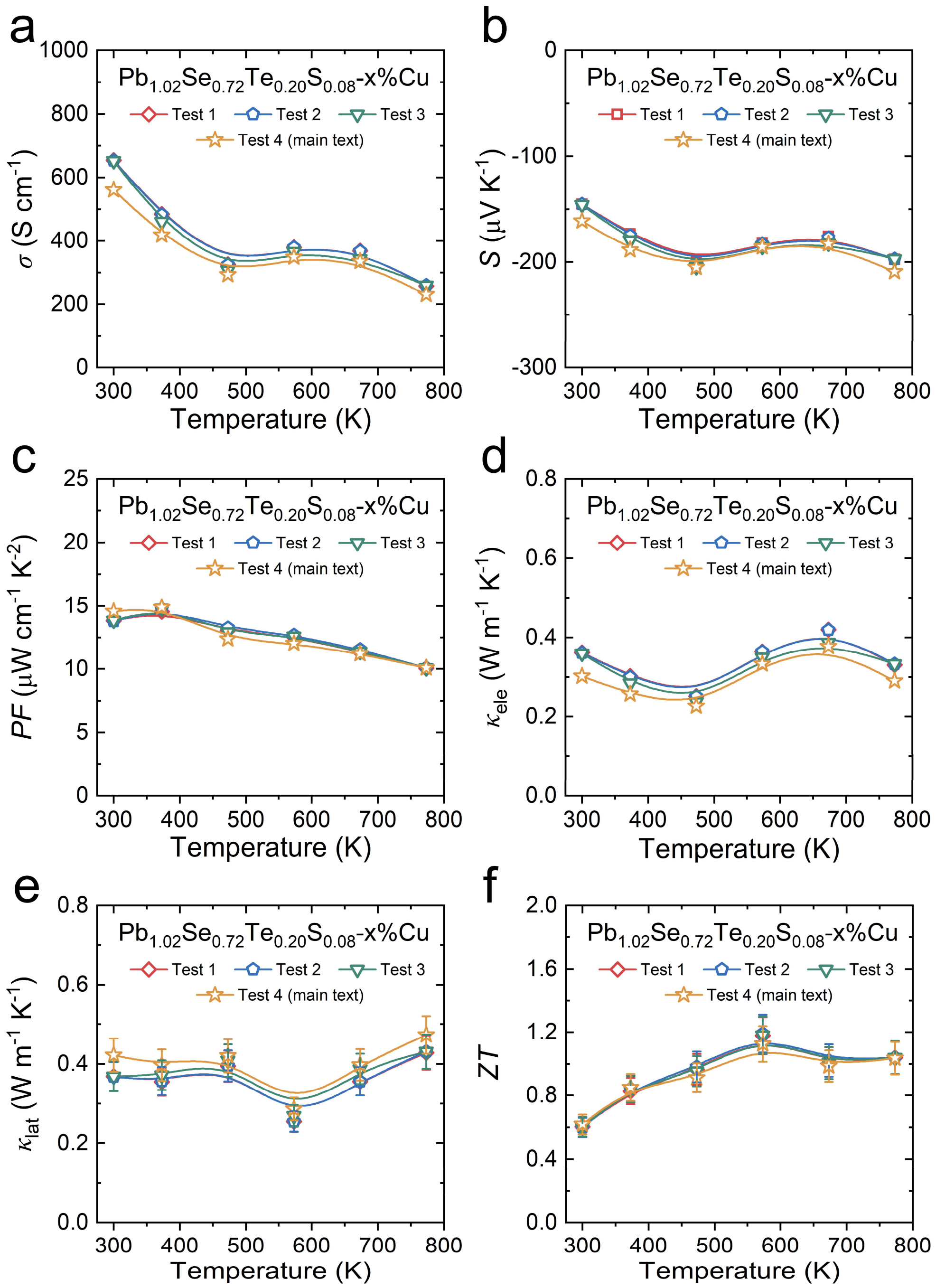

Supplementary Figure 9. Repeatability of the thermoelectric transport properties in $Pb_{1.02}Se_{0.72}Te_{0.20}S_{0.08}$-0.3%Cu: (a) Electrical conductivity. (b) Seebeck coefficient. (c) Power factor. (d) Electronic thermal conductivity. (e) Lattice thermal conductivity. (f) *ZT* values.

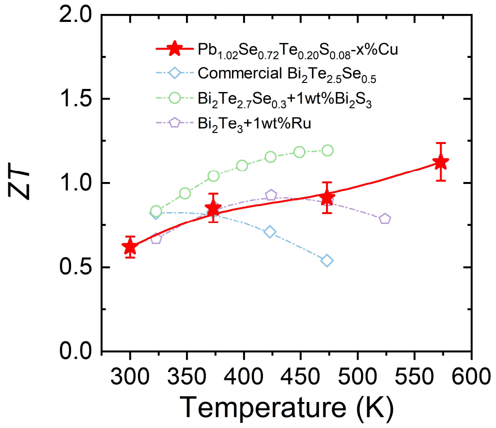

Supplementary Figure 10. Comparison of *ZT* values between $Pb_{1.02}Se_{0.72}Te_{0.20}S_{0.08}$-0.3%Cu and *n*-type Bi_2Te_3-based materials.

Supplementary Table 1. Parameters used for the Callaway model.

Parameters	Description	PbSe	PbTe	PbS	Ref.
v_l	Longitudinal sound velocity (m s^{-1})	3200	2910	3450	[17]
v_s	Shear sound velocity (m s^{-1})	1750	1610	1900	[17]
G	Ratio between the relative change of bulk modulus and banding length	3	3	3	[3, 18-20]

Supplementary Table 2. Values of θ, FWHM, *hkl* and dislocation density in $Pb_{1.02}Se_{0.72}Te_{0.20}S_{0.08}$-x%Cu. The dislocation density is evaluated with a similar Burgers vector ***b*** as observed in TEM results.

Parameters	Samples	Values					
θ (°)	x=0	24.8867	28.8543	41.2968	48.8870	59.8946	75.3682
	x=0.3	24.9752	28.9422	41.3717	48.9716	60.0236	75.4747
(*hkl*)	x=0	(111)	(200)	(220)	(311)	(400)	(422)
	x=0.3	(111)	(200)	(220)	(311)	(400)	(422)
FWHM (°)	x=0	0.4032	0.4067	0.5923	0.6185	0.6710	0.8718
	x=0.3	0.3951	0.3824	0.6074	0.6750	0.7169	1.0359
N_D (m^{-2})	x=0	~ 2.3×10^{16}					
	x=0.3	~ 5.4×10^{16}					

Supplementary Table 3. Parameters used for the modified Williamson-Hall model.

Parameters	Description	Value	Ref.
λ	Wavelength of the synchrotron X-ray (Å)	1.5418	-
A	Parameter determined by the effective outer cut-off radius of dislocations	2.6	[21]
b	Magnitude of Burgers vector (m)	4.15×10^{-10}	This work
$\overline{C}_{h00}$	corresponding to the *h*00 reflection determining by elastic modulus	0.12148	[11, 16]
c_{11}	Elastic modulus (GPa)	123.7	[22]
c_{12}		19.3	
c_{44}		15.9	
O	Non-interpreted higher-order error terms	Non	-

Supplementary References

1. Callaway, J. Model for Lattice Thermal Conductivity at Low Temperatures. *Phys. Rev.* **113**, 1046-1051 (1959).
2. Callaway, J. & von Baeyer, H. C. Effect of Point Imperfections on Lattice Thermal Conductivity. *Phys. Rev.* **120**, 1149-1154 (1960).
3. He, W. et al. High thermoelectric performance in low-cost $SnS_{0.91}Se_{0.09}$ crystals. *Science* **365**, 1418-1424 (2019).
4. Wan, C. L. et al. Effect of point defects on the thermal transport properties of $(La_xGd_{1-x})_2Zr_2O_7$: Experiment and theoretical model. *Phys. Rev. B* **74**, 144109 (2006).
5. Kurosaki, K., Kosuga, A., Muta, H., Uno, M. & Yamanaka, S. Ag_9TlTe_5: A high-performance thermoelectric bulk material with extremely low thermal conductivity. *Appl. Phys. Lett.* **87**, 061919 (2005).
6. Tan, G. et al. SnTe-$AgBiTe_2$ as an efficient thermoelectric material with low thermal conductivity. *J. Mater. Chem. A* **2**, 20849-20854 (2014).
7. Abeles, B. Lattice Thermal Conductivity of Disordered Semiconductor Alloys at High Temperatures. *Phys. Rev.* **131**, 1906-1911 (1963).
8. Wan, C., Qu, Z., He, Y., Luan, D. & Pan, W. Ultralow thermal conductivity in highly anion-defective aluminates. *Phys. Rev. Lett.* **101**, 085901 (2008).
9. Sanditov, D. S. & Belomestnykh, V. N. Relation between the parameters of the elasticity theory and averaged bulk modulus of solids. *Tech. Phys.* **56**, 1619-1623 (2011).
10. Ungár, T. & Borbély, A. The effect of dislocation contrast on x-ray line broadening: A new approach to line profile analysis. *Appl. Phys. Lett.* **69**, 3173-3175 (1996).
11. Ungár, T., Ott, S., Sanders, P. G., Borbély, A. & Weertman, J. R. Weertman. Dislocations, grain size and planar faults in nanostructured copper determined by high resolution X-ray diffraction and a new procedure of peak profile analysis. *Acta Mater.* **46**, 3693-3699 (1998).
12. Williamson, G. K. & Hall, W. H. Hall. X-ray line broadening from filed aluminium and wolfram. *Acta Metall.* **1**, 22-31 (1953).
13. Groma, I., Ungar, T. & Wilkens, M. Asymmetric X-ray line broadening of plastically deformed crystals. I. Theory. *J. Appl. Crystallogr.* **21**, 47-54 (1988).
14. Ungar, T., Groma, I. & Wilkens, M. Asymmetric X-ray line broadening of plastically deformed crystals. II. Evaluation procedure and application to [001]-Cu crystals. *J. Appl. Crystallogr.* **22**, 26-34 (1989).
15. Wilkens, M. The determination of density and distribution of dislocations in deformed single crystals from broadened X-ray diffraction profiles. *Phys. Stat. Sol. (a)* **2**, 359-370 (1970).
16. Ungár, T., Dragomir, I., Révész, Á. & Borbély, A. The contrast factors of dislocations in cubic crystals: the dislocation model of strain anisotropy in practice. *J. Appl. Crystallogr.* **33**, 992-1002 (1999).
17. Xiao, Y. et al. Origin of low thermal conductivity in SnSe. *Phys. Rev. B* **94**, 125203 (2016).
18. Anderson, O. L. & Nafe, J. E. The bulk modulus-volume relationship for oxide compounds and related geophysical problems. *J. Geophys. Res.* **70**, 3951-3963 (1965).
19. Anderson, D. L. & Anderson, O. L. Anderson. Brief report: The bulk modulus-volume relationship for oxides. *J. Geophys. Res.* **75**, 3494-3500 (1970).
20. Wang, H., Wang, J., Cao, X. & Snyder, G. J. Thermoelectric alloys between PbSe and PbS with

effective thermal conductivity reduction and high figure of merit. *J. Mater. Chem. A* **2**, 3169-3174 (2014).

21. Chen, Z. et al. Vacancy-induced dislocations within grains for high-performance PbSe thermoelectrics. *Nat. Commun.* **8**, 13828 (2017).
22. Lippmann, G., Kästner, P. & Wanninger, W. Elastic constants of PbSe. *Phys. Stat. Sol. (a)* **6**, K159-K161 (1971).
